# Estrogenic Activity of Mycoestrogen (3*β*,5*α*,22*E*)-Ergost-22-en-3-ol via Estrogen Receptor α-Dependent Signaling Pathways in MCF-7 Cells

**DOI:** 10.3390/molecules27010036

**Published:** 2021-12-22

**Authors:** Dahae Lee, Yuri Ko, Changhyun Pang, Yoon-Joo Ko, You-Kyoung Choi, Ki Hyun Kim, Ki Sung Kang

**Affiliations:** 1 College of Korean Medicine, Gachon University, Seongnam 13120, Korea; pjsldh@gachon.ac.kr (D.L.); kosmos@gachon.ac.kr (Y.-K.C.); 2 Department of Biological Chemistry and Molecular Pharmacology, Harvard Medical School, Boston, MA 02115, USA; koyr0120@gmail.com; 3 School of Chemical Engineering, Sungkyunkwan University, Suwon 16419, Korea; chpang@skku.edu; 4 Laboratory of Nuclear Magnetic Resonance, National Center for Inter-University Research Facilities (NCIRF), Seoul National University, Gwanak-gu, Seoul 08826, Korea; yjko@snu.ac.kr; 5 School of Pharmacy, Sungkyunkwan University, Suwon 16419, Korea

**Keywords:** *Armillariella tabescens*, (3*β*,5*α*,22*E*)-ergost-22-en-3-ol, phytoestrogens, estrogen receptor

## Abstract

*Armillariella tabescens* (Scop.) Sing., a mushroom of the family *Tricholomataceae*, has been used in traditional oriental medicine to treat cholecystitis, improve bile secretion, and regulate bile-duct pressure. The present study evaluated the estrogen-like effects of *A. tabescens* using a cell-proliferation assay in an estrogen-receptor-positive breast cancer cell line (MCF-7). We found that the methanol extract of *A. tabescens* fruiting bodies promoted cell proliferation in MCF-7 cells. Using bioassay-guided fractionation of the methanol extract and chemical investigation, we isolated and identified four steroids and four fatty acids from the active fraction. All eight compounds were evaluated by E-screen assay for their estrogen-like effects in MCF-7 cells. Among the tested isolates, only (3*β*,5*α*,22*E*)-ergost-22-en-3-ol promoted cell proliferation in MCF-7 cells; this effect was mitigated by the ER antagonist, ICI 182,780. The mechanism underlying the estrogen-like effect of (3*β*,5*α*,22*E*)-ergost-22-en-3-ol was evaluated using Western blot analysis to detect the expression of extracellular signal-regulated kinase (ERK), phosphatidylinositol 3-kinase (PI3K), Akt, and estrogen receptor α (ERα). We found that (3*β*,5*α*,22*E*)-ergost-22-en-3-ol induced an increase in phosphorylation of ERK, PI3K, Akt, and ERα. Together, these experimental results suggest that (3*β*,5*α*,22*E*)-ergost-22-en-3-ol is responsible for the estrogen-like effects of *A. tabescens* and may potentially aid control of estrogenic activity in menopause.

## 1. Introduction

**Figure 1:**
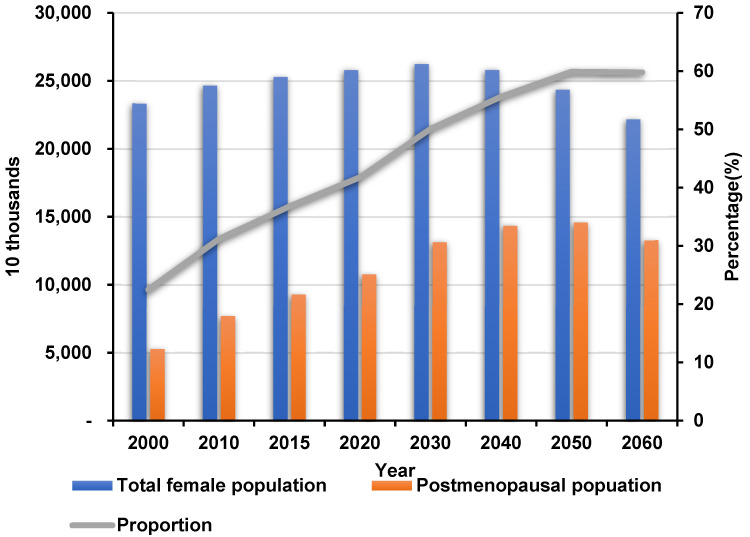
Transitions of the total female and postmenopausal female populations in South Korea. The total female population will peak in 2030 then decrease through to 2060. The postmenopausal female population will continuously increase. After 2030, over half of South Korean females will be postmenopausal [3].

Menopause, the complete end of menstrual periods, typically occurs worldwide for women 45 to 55 years of age. With increasing life expectancy, the global number of menopausal women aged 50 years and over is estimated to reach 1.2 billion by 2030, with 47 million new entrants each year [1,2]. In South Korea, the postmenopausal female population aged over 50 years has increased since 2000. After 2030, over half of the female population will be postmenopausal (Figure 1), according to the Korean Statistical Information Service database [3]. Menopause results from declining ovarian function, and the production of steroid hormones such as estrogen dramatically drops. As a result, menopause results in such vasomotor symptoms as hot flushes, night sweats, sleep disturbance, vaginal dryness, and even osteoporosis [4]. Women experiencing menopausal symptoms have reported significantly reduced health-related quality of life; in the most severe cases, exogenous estrogens are prescribed [5].

Estrogen replacement therapy has traditionally been considered beneficial for the relief and prevention of postmenopausal symptoms and related diseases [6,7]. However, patients receiving estrogen replacement therapy over the long term are often reluctant to continue because of side effects including breast cancer, heart disease, and stroke [8,9]. Thus, there is growing interest in the use of phytoestrogens with estrogen-like activity. Phytoestrogens are natural compounds found in plants and plant-based foods and are structurally, and sometimes functionally, similar to mammalian estrogens and their active metabolites [10]. Phytoestrogens such as stilbenes, isoflavonoids, lignans, and flavonoids are reported to be abundant in red clover plants, flax seeds, and soy plants [11]. When phytoestrogens bind to the estrogen receptor (ER), they act as agonists or antagonists [12]. They are structurally similar to estrogen; in theory, therefore, they can increase the risk of breast cancer development [7]. However, some studies on the effects of phytoestrogens on breast cancer have suggested that phytoestrogens exhibit no effect on cancer or even exhibit anti-cancer effects [13].

Mycoestrogens, natural fungus-derived products with estrogen-like activity, can be produced by various *Fusarium* species [14]. Mycoestrogens have features similar to those of phytoestrogens and are reported to act as estrogen-receptor agonists [15]. Mushrooms—the fleshy, spore-bearing fruiting bodies of fungi—have been used as functional foods and dietary supplements because of various bioactive secondary metabolites that exhibit interesting biological actions [16]. As an example of the estrogen-like activity of a mushroom, the ethanol extract of the *Pleurotus eryngii* fruiting body is well documented to exhibit proliferative effects in ER-positive MCF-7 human breast epithelial cell lines and to promote ovariectomy-induced bone loss in old female rats [17]. However, the chemical contributors to the estrogen-like effects of *P. eryngii* have not yet been identified.

Our group has been conducting extended natural-product research to discover bioactive compounds from Korean wild mushrooms [18,19,20,21,22,23,24,25]. In this context, we investigated potential bioactive compounds from a methanol (MeOH) extract of the fruiting bodies of *Armillariella* *tabescens* (Scop.) Sing. to show anti-gastritic activity against ethanol-induced gastric damage in rats. The previous study found that (*Z*,*Z*)-9,12-octadecadienoic acid, isolated from *A. tabescens* as a fatty acid, exhibited anti-inflammatory activity involved in anti-gastritic activity [23]. *A. tabescens*, belonging to the *Tricholomataceae* family, is known as the “Luminous Fungus” in China. This mushroom has been used in traditional medicine to treat cholecystitis, to regulate bile-duct pressure, and to improve liver function [26,27]. Previous studies of *A. tabescens* extracts have reported that the extracts exhibit antitumor and immunomodulatory activities [23,28,29]. However, few studies of *A. tabescens* have investigated its chemical constituents, despite the potential pharmacological applications. Previous chemical investigations of *A. tabescens* have reported armillarisins A and B, which exhibited biological activities including antifungal effects and anti-infection properties against gastritis and hepatitis [26,27]. In our ongoing research on *A. tabescens,* we found that the MeOH extract of the fruiting bodies of *A. tabescens* showed estrogen-like effects in the estrogen-receptor-positive MCF-7 breast cancer cell line. The estrogen-like effect of *A. tabescens* has not previously been reported. Thus, the present study was conducted to further investigate the active MeOH extracts to identify potential mycoestrogens. Herein, we describe the isolation and structural characterization of eight compounds and evaluate their estrogen-like effects in MCF-7 cells; we also characterize the bioactivity of the active compound as a mycoestrogen.

## 2. Results

### 2.1. Bioactivity-Guided Fractionation of the MeOH Extract of A. tabescens

**Figure 2:**
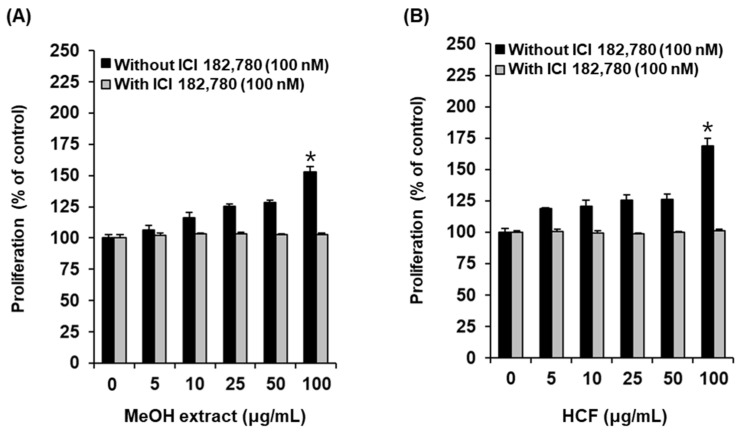
Comparison of estrogenic activities of (**A**) MeOH extract and (**B**) hexane- and CH_2_Cl_2_-soluble fraction (HCF) in the absence or presence of ICI 182,780, as determined by cell proliferation measured by E-screen assay in MCF-7 cells. * Significant difference between untreated cells and cells treated with sample in the absence of ICI 182,780 (*n* = 3 independent experiments, *p* < 0.05, Kruskal–Wallis nonparametric test). Data are the mean ± SEM.

We examined MCF-7 cell proliferation after treatment with the MeOH extract of *A. tabescens* using Ez-Cytox reagents. Cell proliferation increased to 152.61 ± 4.73% after treatment, with 100 µg/mL of the MeOH extract compared with the untreated cells, and this effect was mitigated by ICI 182,780, an estrogen receptor (ER) antagonist (Figure 2A). Based on this result, the MeOH extract was successively solvent partitioned with hexane, CH_2_Cl_2_, EtOAc, and *n*-BuOH to give four main fractions: hexane-soluble, CH_2_Cl_2_-soluble, EtOAc-soluble, and *n*-BuOH-soluble. LC/MS and TLC analyses indicated that the hexane-soluble and CH_2_Cl_2_-soluble fractions had similar chemical profiles, which allowed us to consolidate the hexane- and CH_2_Cl_2_-soluble fractions, yielding the HCF for further experiments. For the bioactivity-guided fractionation of the MeOH extract, the estrogen-like effects of the three main fractions (HCF, EtOAc-soluble, and *n*-BuOH-soluble) were evaluated using a cell-proliferation assay to identify the active fraction. Of the fractions tested, cell proliferation increased to 169.01 ± 5.91% after treatment with the HCF fraction compared with the untreated cells, and this effect was mitigated by the ICI 182,780 (Figure 2B).

### 2.2. Isolation and Identification of Compounds from the Active Fraction

**Figure 3:**
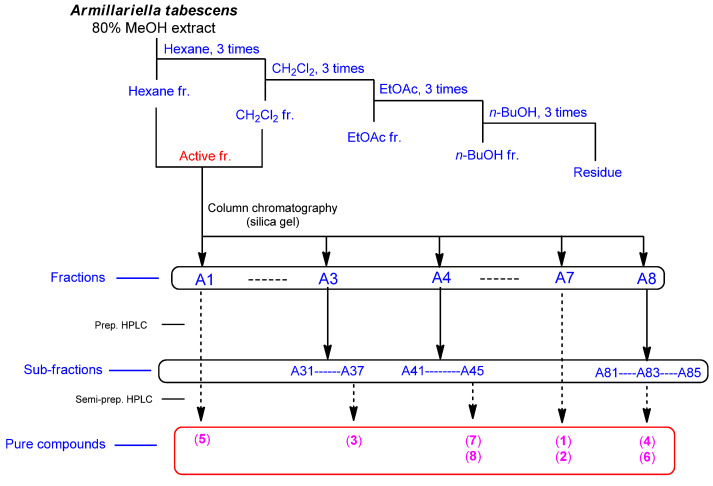
Separation scheme of compounds **1**–**8**.

**Figure 4:**
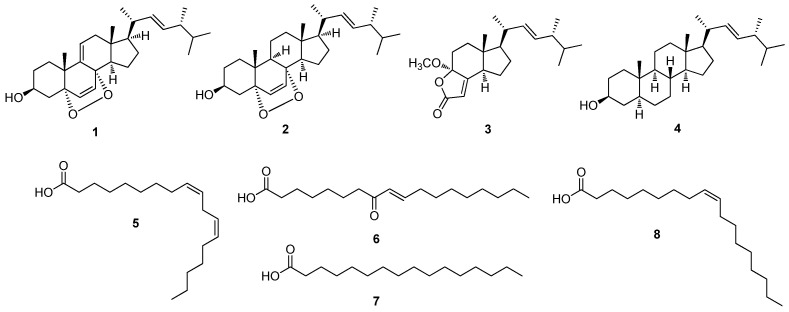
Chemical structures of compounds **1**–**8**; 9,11-dehydroergosterol peroxide (**1**), ergosterol peroxide (**2**), (17*R*)-17-methylincisterol (**3**), (3*β*,5*α*,22*E*)-ergost-22-en-3-ol (**4**), (*Z*,*Z*)-9,12-octadecadienoic acid (**5**), (9*E*)-8-oxo-9-octadecenoic acid (**6**), hexadecanoic acid (**7**), and (9*Z*)-9-octadecenoic acid (**8**).

Based on the results of the bioactivity-guided fractionation for estrogen-like effects, chemical investigation of the active HCF fraction was conducted to identify the chemical contributors to the estrogenic activity of the MeOH extract of *A. tabescens*. Chemical investigation of the EA fraction, using column chromatography and preparative and semi-preparative high-performance liquid chromatography (HPLC) purification, led to the isolation and identification of four steroids (**1**–**4**) and four fatty acids (**5**–**8**) from the active fraction (Figure 3). The structures of compounds **1**–**8** (Figure 4) were determined to be 9,11-dehydroergosterol peroxide (**1**) [30], ergosterol peroxide (**2**) [30], (17*R*)-17-methylincisterol (**3**) [31], (3*β*,5*α*,22*E*)-ergost-22-en-3-ol (**4**) [32], (*Z*,*Z*)-9,12-octadecadienoic acid (**5**) [33], (9*E*)-8-oxo-9-octadecenoic acid (**6**) [34], hexadecanoic acid (**7**) [35], and (9*Z*)-9-octadecenoic acid (**8**) [36], by comparing their ^1^H and ^13^C NMR spectra (Figures S1–S9) with those previously reported in the literature and by LC/MS analysis (Figures S10–S17).

### 2.3. Effects of Compounds on the Proliferation of MCF-7 Cells

**Figure 5:**
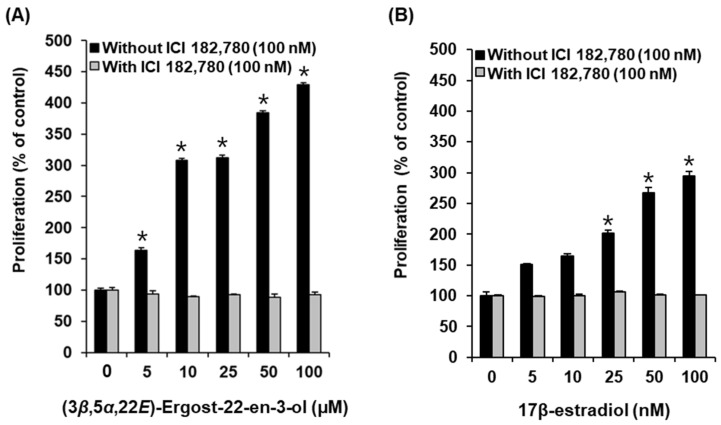
Comparison of estrogenic activities of (**A**) (3*β*,5*α*,22*E*)-ergost-22-en-3-ol (**4**) and (**B**) 17β-estradiol (E2) in the absence or presence of ICI 182,780, as determined by cell proliferation measured by E-screen assay in MCF-7 cells. * Significant difference between untreated cells and cells treated with sample in the absence of ICI 182,780 (*n* = 3 independent experiments, *p* < 0.05, Kruskal–Wallis nonparametric test). Data are the mean ± SEM.

All the isolated compounds **1**–**8** were tested for their effects on MCF-7 cell proliferation to investigate their estrogenic activity. Among the tested isolates **1**–**8**, only (3*β*,5*α*,22*E*)-ergost-22-en-3-ol (compound **4**) promoted cell proliferation in MCF-7 cells. Cell proliferation increased to 163.23 ± 4.23%, 384.38 ± 3.07%, and 429.33 ± 2.52% after treatment with 25 µM, 50 µM, and 100 µM of compound **4**, respectively, and the effects were mitigated by the ICI 182,780, an ER antagonist (Figure 5A). Cell proliferation increased to 201.71 ± 4.47%, 266.82 ± 8.72%, and 294.87 ± 7.31% after treatment with 25 nM, 50 nM, and 100 nM, respectively, of 17β-estradiol (E2) as a positive control, compared with the untreated cells (Figure 5B). These results proved that compound **4** is an effective phytoestrogen demonstrating E2-like activity in the proliferation of estrogen-receptor-positive breast cancer cells.

### 2.4. Effect of (3β,5α,22E)-Ergost-22-en-3-ol on the Protein Expression of Phospho-PI3K, PI3K, Phospho-Akt, Akt, Phospho-ERα, and ERα

**Figure 6:**
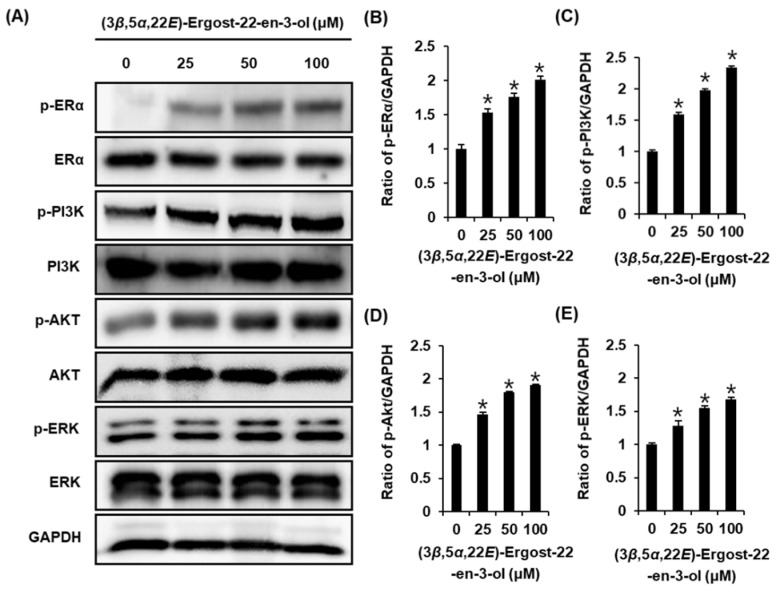
Effect of (3*β*,5*α*,22*E*)-ergost-22-en-3-ol (compound **4**) on the protein expression of phospho-extracellular-signal-regulated kinase (p-ERK), ERK, phospho-phosphatidylinositol 3-kinase (p-PI3K), PI3K, phospho-Akt (p-Akt), Akt, phospho-estrogen receptor α (p-ERα), and ERα in MCF-7 cells. (**A**) Protein expression levels of p-PI3K, PI3K, p-Akt, Akt, p-ERα, ERα, and glyceraldehyde 3-phosphate dehydrogenase (GAPDH) in MCF-7 cells treated or untreated with 25 µM, 50 µM, and 100 µM compound **4** for 24 h. (**B**–**E**) Bar graph presents the densitometric quantification of Western blot bands. * Significant difference between cells treated with compound **4** and the untreated cells (*n* = 3 independent experiments, *p* < 0.05, Kruskal–Wallis nonparametric test). Data are the mean ± SEM.

**Figure 7:**
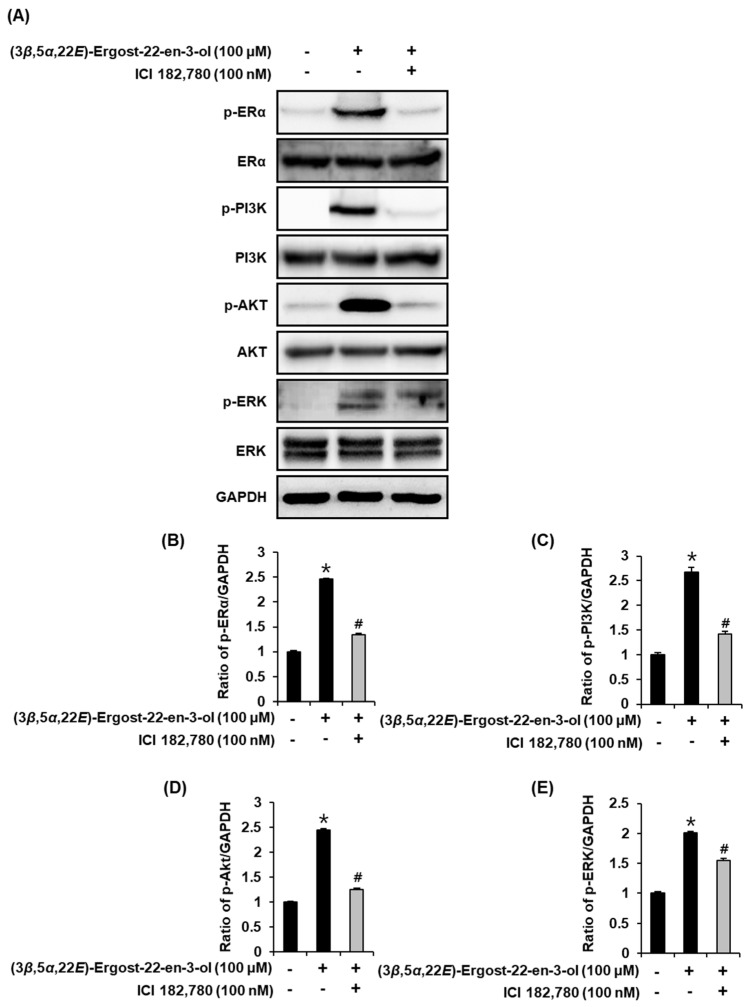
Effect of (3*β*,5*α*,22*E*)-ergost-22-en-3-ol (compound **4**) in the absence or presence of ICI 182,780 (ICI) on the protein expression of phospho-extracellular-signal-regulated kinase (p-ERK), ERK, phospho-phosphatidylinositol 3-kinase (p-PI3K), PI3K, phospho-Akt (p-Akt), Akt, phospho-estrogen receptor α (p-ERα), and ERα in MCF-7 cells. (**A**) Protein expression levels of p-PI3K, PI3K, p-Akt, Akt, p-ERα, ERα, and glyceraldehyde 3-phosphate dehydrogenase (GAPDH) in MCF-7 cells treated or untreated with concentrations of 100 µM compound **4**, either with or without 100 nM ICI 182,780 (ICI) for 24 h. (**B**–**E**) Bar graph presents the densitometric quantification of Western blot bands. * Significant difference between cells treated with compound **4** and the untreated cells. # Significant reduction in co-treatment with ICI compared to treatment with compound **4** alone (*n* = 3 independent experiments, *p* < 0.05, Kruskal–Wallis nonparametric test). Data are represented as mean ± SEM.

**Figure 8:**
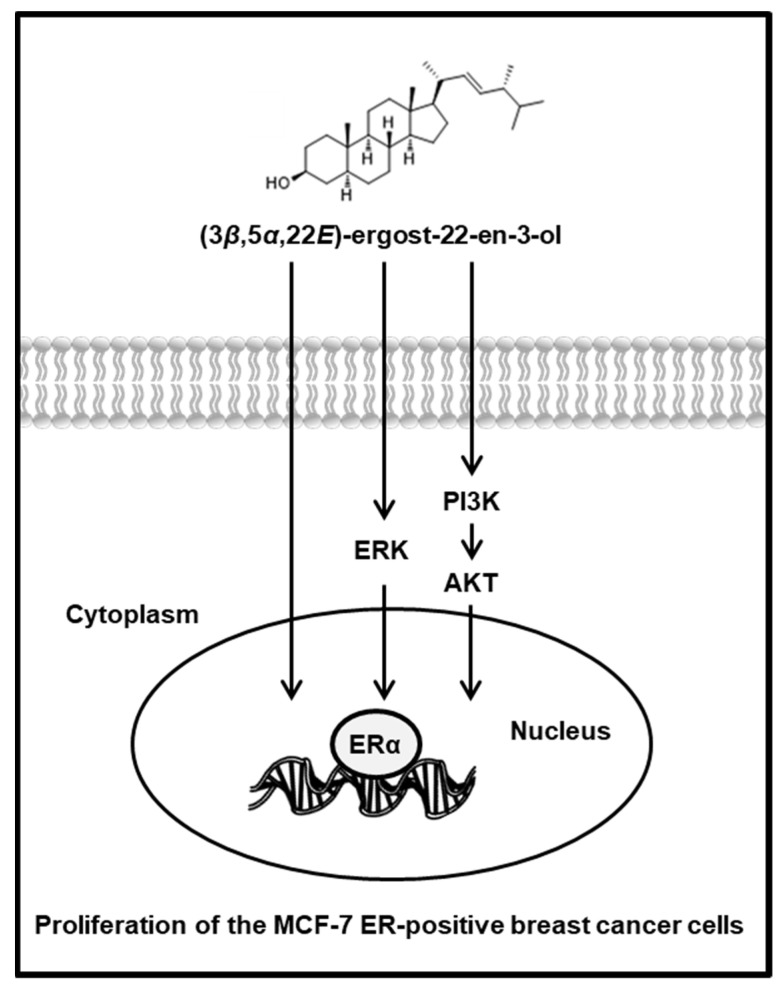
Schematic illustration of the underlying mechanism of the estrogenic activity of (3*β*,5*α*,22*E*)-ergost-22-en-3-ol via estrogen receptor α (ERα)-dependent signaling pathways in MCF-7 estrogen-receptor-positive breast cancer cells.

To support the proliferation-promoting effects of (3*β*,5*α*,22*E*)-ergost-22-en-3-ol (**4**), the activation of ERα and related pathways were evaluated using Western blot analysis. Compared with untreated cells, 25 µM, 50 µM, and 100 µM of compound **4** induced a concentration-dependent increase in the protein expression of p-ERK, p-PI3K, p-Akt, and p-ERα (Figure 6). Furthermore, this effect was mitigated by treatment with 100 nM ICI. When ICI was present, the expression of p-ERK, p-PI3K, p-Akt, and ERα failed to increase after treatment with 100 µM of compound **4** (Figure 7). These results proved that the responses of ERK, PI3K, and Akt to compound **4** depend on the functioning of ER (Figure 8).

## 3. Discussion

Few previous studies have investigated the compounds isolated from *A. tabescens*. Our previous chemical studies on *A. tabescens* have shown the presence of steroids, alkaloids, nucleic acids, and fatty acids. (Z,Z)-9,12-Octadecadienoic acid, a fatty acid, is known to possess anti-inflammatory activity [23]. Extending from our previous study, the present study found that the MeOH extract of *A. tabescens* had estrogen-like effects on MCF-7 cells. Eight compounds were isolated from the active fraction, the hexane- and CH_2_Cl_2_-soluble fraction showing estrogen-like effects. Of the eight compounds tested, only (3*β*,5*α*,22*E*)-ergost-22-en-3-ol (compound **4**) promoted cell proliferation in MCF-7 cells. Co-treatment with ICI 182,780, an ER antagonist, inhibited the proliferation-stimulatory effect. These results indicated that compound **4** exhibited a proliferation-stimulatory effect via the ER in MCF-7 cells. Its proliferation-stimulatory effect was confirmed by the expression of proteins related to the ER signaling pathway. The binding of estrogen to the G-protein-coupled estrogen receptor (GPER) activates the ERK and PI3K/Akt pathways [37,38]. ERK is a family of mitogen-activated protein kinases (MAPKs) stimulated by peptide hormones, cellular stress, and cytokines. It regulates the proliferation of ER-positive breast cancer cells [39]. The activated PI3K/Akt pathway regulates cellular growth, survival, and proliferation in normal estrogen-responsive tissues [40,41]. Ginsenoside Rg1, a chemical component of ginseng, has been reported to possess estrogen-like effects and promote ER signaling via the ERK and PI3K/Akt pathways [42]. Various studies have reported the estrogen-like effect of acacetin, a flavonoid, and its possible mechanism has also been evaluated as the ERK and PI3K/Akt pathway [43,44,45]. In our study, (3*β*,5*α*,22*E*)-ergost-22-en-3-ol also induced a concentration-dependent increase in the protein expression of p-ERK, p-PI3K, p-Akt, and p-ERα in MCF-7 cells, similar to that of other reported phytoestrogens. Therefore, it was concluded that the estrogen-like effect of (3*β*,5*α*,22*E*)-ergost-22-en-3-ol was mainly mediated via ERα. These cell-based results demonstrated for the first time that the extract of *A. tabescens* exhibited potent estrogen-like effects. Among the isolates, (3*β*,5*α*,22*E*)-ergost-22-en-3-ol was the main contributor to the estrogen-like effect of *A. tabescens*; it may be a potential candidate for further verification in animal experiments, towards finding estrogen-like drugs to eventually help postmenopausal women.

## 4. Materials and Methods

### 4.1. General Experimental Procedures

Optical rotations were measured using a Jasco P-2000 polarimeter (Jasco, Easton, MD, USA). Ultraviolet (UV) spectra were acquired using an Agilent 8453 UV-visible spectrophotometer (Agilent Technologies, Santa Clara, CA, USA). NMR spectra were measured using a Bruker AVANCE III 700 NMR spectrometer operating at 700 MHz (^1^H) and 175 MHz (^13^C; Bruker, Billerica, MA, USA). Preparative high-performance liquid chromatography (HPLC) was performed using a Waters 1525 Binary HPLC Pump with a Waters 996 Photodiode Array Detector (Waters Corporation, Milford, CT, USA) and an Agilent Eclipse C18 column (250 × 21.2 mm, 5 μm; flow rate: 5 mL/min; Agilent Technologies, Santa Clara, CA, USA). Semi-preparative HPLC was performed using a Shimadzu Prominence HPLC System with SPD-20A/20AV Series Prominence HPLC UV-Vis detectors (Shimadzu, Tokyo, Japan) and a Phenomenex Luna phenyl-hexyl column (250 × 10 mm inner diameter [ID], flow rate: 2 mL/min; Phenomenex, Torrance, CA, USA). Liquid chromatography–mass spectrometry (LC/MS) analysis was performed using an Agilent 1200 Series HPLC system equipped with a diode array detector and 6130 Series ESI mass spectrometer using an analytical Kinetex C18 100 Å column (100 × 2.1 mm, 5 μm; flow rate: 0.3 mL/min; Phenomenex, Torrance, CA, USA). Column chromatography was performed using silica gel 60, 230–400 mesh, and reverse-phase (RP) C18 silica gel, 230–400 mesh (Merck, Darmstadt, Germany). Sephadex LH-20 (Pharmacia, Uppsala, Sweden) was used for molecular-sieve column chromatography. Thin-layer chromatography (TLC) was conducted using precoated silica gel F_254_ plates and RP-18 F_254s_ plates (Merck). Spots on TLC were detected using UV light and heating after dipping in anisaldehyde sulfuric acid.

### 4.2. Fungus Material

Fresh fruiting bodies of *A. tabescens* were collected from Hwasung, Gyeonggi-do, Korea, in September 2014. Samples of fungal material were identified by one of the authors (K.H.K.). A voucher specimen (SKKU 2015-09-BN) was deposited in the herbarium of the School of Pharmacy, Sungkyunkwan University, Suwon, Korea.

### 4.3. Extraction and Isolation

Shade-dried and chopped *A. tabescens* mushrooms (310 g) were extracted with 80% aqueous MeOH three times (each 3 L × 24 h) at room temperature. The resulting extracts were filtered, and the filtrate concentrated under reduced pressure using a rotary evaporator (EYELA, Tokyo Rikakikai Co., Tokyo, Japan). The resultant MeOH extract (28.7 g) was suspended in distilled water (700 mL) and successively solvent-partitioned three times using hexane, dichloromethane (CH_2_Cl_2_), ethyl acetate (EtOAc), and *n*-butanol (*n*-BuOH), which yielded a hexane-soluble fraction (0.9 g), a CH_2_Cl_2_-soluble fraction (1.0 g), an EtOAc-soluble fraction (0.3 g), and an *n*-BuOH-soluble fraction (1.7 g). LC/MS and TLC analyses indicated that the hexane-soluble and CH_2_Cl_2_-soluble layers had similar chemical profiles, allowing us to consolidate the hexane- and CH_2_Cl_2_-soluble layers for further experiments. The active hexane- and CH_2_Cl_2_-soluble fraction (HCF; 1.9 g) was subjected to silica-gel (230–400 mesh, Merck, Kenilworth, NJ, USA) column chromatography (CC), using a gradient solvent system of hexane/EtOAc from 30:1 to 1:1, to yield eight fractions (Fr. A1–A8). Fr. A1 (353 mg) was purified by semi-preparative HPLC (solvent system of 91% MeOH) using a Phenomenex Luna phenyl-hexyl column (250 × 10 mm ID, flow rate: 2 mL/min) to isolate compound **5** (13.1 mg). Fr. A3 (192 mg) was separated by preparative HPLC (solvent system of 83% MeOH) using an Agilent Eclipse C18 column (250 × 21.2 mm ID, flow rate: 5 mL/min) to obtain seven fractions (Fr. A31–A37). Fr. A37 (85 mg) was further purified by semi-preparative HPLC (solvent system of 88% MeOH) using the Phenomenex Luna phenyl-hexyl column system to yield compound **3** (1.7 mg). Fr. A4 (174 mg) was fractionated with preparative HPLC (gradient solvent system of 85–100% MeOH), using the above conditions of the Agilent Eclipse C18 column, to give five fractions (Fr. A41–A45). Fr. A45 (94 mg) was further purified using preparative HPLC (solvent system of 84% MeOH) with the same column to obtain five subfractions (Fr. A451–A455). Fr. A455 (53 mg) was separated by semi-preparative HPLC (solvent system of 87% MeOH) using a Phenomenex Luna phenyl-hexyl column, which yielded compounds **7** (7.3 mg) and **8** (2.4 mg). Fr. A7 (24.6 mg) was directly subjected to semi-preparative HPLC (solvent system of 90% MeOH) using a Phenomenex Luna phenyl-hexyl column to purify compounds **1** (0.9 mg) and **2** (1.2 mg). Fr. A8 (380 mg) was separated on a Sephadex LH-20 column using a solvent system of CH_2_Cl_2_/MeOH (2:8), and five fractions were obtained (Fr. A81–A85). Fr. A83 (155 mg) was separated by preparative HPLC (gradient solvent system of 70–100% MeOH) using an Agilent Eclipse C18 column to give five subfractions (Fr. A831–A835). Fr. A834 (14.8 mg) was further purified using semi-preparative HPLC (solvent system of 76% MeOH) using a Phenomenex Luna phenyl-hexyl column to yield compound **6** (0.9 mg), and Fr. A835 (24 mg) was also purified by semi-preparative HPLC (solvent system of 85% MeOH) using the same column to yield compound **4** (1.0 mg).

### 4.4. Cell Culture

The ER-positive MCF-7 human breast epithelial cell line (American Type Culture Collection, Manassas, VA, USA) was cultured in Roswell Park Memorial Institute-1640 (RPMI1640) medium (Cellgro, Manassas, VA, USA) with 100 μg/mL streptomycin, 100 U/mL penicillin, and 10% fetal bovine serum (Gibco BRL, Grand Island, NY, USA). MCF-7 cells were stored at 37 °C in an incubator with a CO_2_ concentration of approximately 5%.

### 4.5. E-Screen Assay

MCF-7 cells were cultured in 24-well plates to a final concentration of 1 × 10^5^ cells per well in RPMI medium without phenol red (Gibco BRL, Grand Island, NY, USA) with 100 μg/mL streptomycin, 100 U/mL penicillin, and 5% charcoal-dextran stripped human serum (Innovative Research, Novi, MI, USA) for 24 h. MCF-7 cells were treated with MeOH extract (5–100 μg/mL), HCF (5–100 μg/mL), the isolated compounds **1**–**8** (5–100 μM), and 17β-estradiol (E2; 5–100 nM), either with or without ICI 182,780 (100 nM), for 144 h. Ez-Cytox reagents (Daeil Lab Service Co., Seoul, Korea) were added to each well and incubated for 40 min. The absorbance of each well was read at 450 nm using a microplate reader (PowerWave XS).

### 4.6. Western Blot Analysis

MCF-7 cells were cultured in 6-well plates at a final concentration of 4 × 10^5^ cells per well in RPMI medium without phenol red (Gibco BRL, Grand Island, NY, USA) with 100 μg/mL streptomycin, 100 U/mL penicillin, and 5% charcoal-dextran stripped human serum (Innovative Research, Novi, MI, USA) for 24 h. MCF-7 cells were treated with compound **4** (25 μM to 100 μM) for 24 h. Proteins (20 µg) from MCF-7 cells were fractionated by SDS-PAGE (10% polyacrylamide gel) and transferred onto a polyvinylidene fluoride (PVDF) membrane. Phospho-extracellular signal-regulated kinase (p-ERK), ERK, phospho-phosphatidylinositol 3-kinase (p-PI3K), PI3K, phospho-Akt, Akt, phospho-estrogen receptor α (p-ERα), ERα, and glyceraldehyde-3-phosphate dehydrogenase (GAPDH; Cell Signaling Technology) were used as primary antibodies. Protein bands were visualized on a FUSION Solo Chemiluminescence System (PEQLAB Biotechnologie GmbH, Erlangen, Germany) using ECL Advance Western blotting detection reagents (GE Healthcare, Little Chalfont, UK).

### 4.7. Statistical Analysis

All experiments were performed in triplicate. All analyses were performed using SPSS Statistics ver. 19.0 (SPSS Inc., Chicago, IL, USA). Non-parametric comparisons of samples were conducted using the Kruskal–Wallis test to analyze the results. Differences were considered statistically significant at *p* < 0.05.

## 5. Conclusions

In this study, we reported the estrogenic effect of (3*β*,5*α*,22*E*)-ergost-22-en-3-ol isolated from the MeOH extract of the fruiting bodies of *A. tabescens* in MCF-7 estrogen-receptor-positive breast cancer cells. Our results demonstrated that (3*β*,5*α*,22*E*)-ergost-22-en-3-ol significantly increased the proliferation of MCF-7 cells, which was associated with the activation of ERK, PI3K, Akt, and ERα. (3*β*,5*α*,22*E*)-Ergost-22-en-3-ol may have potential for use in controlling the estrogenic activity involved in menopausal symptoms.

## Supplementary Materials

The following supporting information can be downloaded online. Figure S1: The ^1^H NMR spectrum of **1** (CDCl_3_, 700 MHz), Figure S2: The ^1^H NMR spectrum of **2** (CDCl_3_, 700 MHz), Figure S3: The ^1^H NMR spectrum of **3** (CDCl_3_, 700 MHz), Figure S4: The ^13^C NMR spectrum of **3** (CDCl_3_, 175 MHz), Figure S5: The ^1^H NMR spectrum of **4** (CD_3_OD, 700 MHz), Figure S6: The ^1^H NMR spectrum of **5** (CDCl_3_, 700 MHz), Figure S7: The ^1^H NMR spectrum of **6** (CDCl_3_, 700 MHz), Figure S8: The ^1^H NMR spectrum of **7** (CDCl_3_, 700 MHz), Figure S9: The ^1^H NMR spectrum of **8** (CDCl_3_, 700 MHz), Figure S10: Total ion chromatogram (TIC) of compound **1** in the LC/MS analysis, Figure S11: TIC of compound **2** in the LC/MS analysis, Figure S12: TIC of compound **3** in the LC/MS analysis, Figure S13: TIC of compound **4** in the LC/MS analysis, Figure S14: TIC of compound **5** in the LC/MS analysis, Figure S15: TIC of compound **6** in the LC/MS analysis, Figure S16: TIC of compound **7** in the LC/MS analysis, Figure S17: TIC of compound **8** in the LC/MS analysis, Figure S18: UV chromatogram of LC/MS (detection wavelength was set as 254 nm) of (A) hexane-soluble and (B) CH_2_Cl_2_-soluble factions, Figure S19: TLC analysis of (A) hexane-soluble and (B) CH_2_Cl_2_-soluble factions.

## Sample Availability

Samples of the compounds are not available from the authors.
