# Supplementary material for: Estrogenic Activity of Mycoestrogen (3β,5α,22E)-Ergost-22-en-3-ol via Estrogen Receptor α-Dependent Signaling Pathways in MCF-7 Cells
Source: Molecules. 2021 Dec 22;27(1):36. doi: 10.3390/molecules27010036 (PMC8746416; doi:10.3390/molecules27010036)

Supplementary Materials

Estrogenic Activity of Mycoestrogen (3*β*,5*α*,22*E*)-Ergost-22-en-3-ol via Estrogen Receptor *α*-Dependent Signaling Pathways in MCF-7 cells

Dahae Lee [1,†], Yuri Ko [2,†], Changhyun Pang [3], Yoon-Joo Ko [4], You-Kyoung Choi [1], Ki Hyun Kim [2,5,*] and Ki Sung Kang [1,*]

[1] College of Korean Medicine, Gachon University, Seongnam 13120, Korea; pjsldh@gachon.ac.kr (D.L.); kosmos@gachon.ac.kr (Y-K.C.);

[2] Department of Biological Chemistry and Molecular Pharmacology, Harvard Medical School, Boston, MA, USA; koyr0120@gmail.com (Y.K.)

[3] School of Chemical Engineering, Sungkyunkwan University, Suwon 16419, Korea; chpang@skku.edu (C.P.)

[4] Laboratory of Nuclear Magnetic Resonance, National Center for Inter-University Research Facilities (NCIRF), Seoul National University, Gwanak-gu, Seoul 08826, Korea; yjko@snu.ac.kr (Y.J.K.)

[5] School of Pharmacy, Sungkyunkwan University, Suwon 16419, Korea

* Correspondence: kkang@gachon.ac.kr (K.S.K.); khkim83@skku.edu (K.H.K.); Tel.: +82-31-750-5402 (K.S.K.); +82-31-290-7700 (K.H.K.)

† These authors contributed equally to this study

Supplementary Materials Contents:

Figure S1: The ^{1}H NMR spectrum of **1** ($CDCl_3$, 700 MHz), Figure S2: The ^{1}H NMR spectrum of **2** ($CDCl_3$, 700 MHz), Figure S3: The ^{1}H NMR spectrum of **3** ($CDCl_3$, 700 MHz), Figure S4: The ^{13}C NMR spectrum of **3** ($CDCl_3$, 175 MHz), Figure S5: The ^{1}H NMR spectrum of **4** (CD_3OD, 700 MHz), Figure S6: The ^{1}H NMR spectrum of **5** ($CDCl_3$, 700 MHz), Figure S7: The ^{1}H NMR spectrum of **6** ($CDCl_3$, 700 MHz), Figure S8: The ^{1}H NMR spectrum of **7** ($CDCl_3$, 700 MHz), Figure S9: The ^{1}H NMR spectrum of **8** ($CDCl_3$, 700 MHz), Figure S10: Total ion chromatogram (TIC) of compound **1** in the LC/MS analysis, Figure S11: TIC of compound **2** in the LC/MS analysis, Figure S12: TIC of compound **3** in the LC/MS analysis, Figure S13: TIC of compound **4** in the LC/MS analysis, Figure S14: TIC of compound **5** in the LC/MS analysis, Figure S15: TIC of compound **6** in the LC/MS analysis, Figure S16: TIC of compound **7** in the LC/MS analysis, Figure S17: TIC of compound **8** in the LC/MS analysis, Figure S18: UV chromatogram of LC/MS (detection wavelength was set as 254 nm) of (A) hexane-soluble and (B) CH_2Cl_2-soluble factions, Figure S19: TLC analysis of (A) hexane-soluble and (B) CH_2Cl_2-soluble factions.

Figure S1. The ^{1}H NMR spectrum of **1** ($CDCl_3$, 700 MHz)

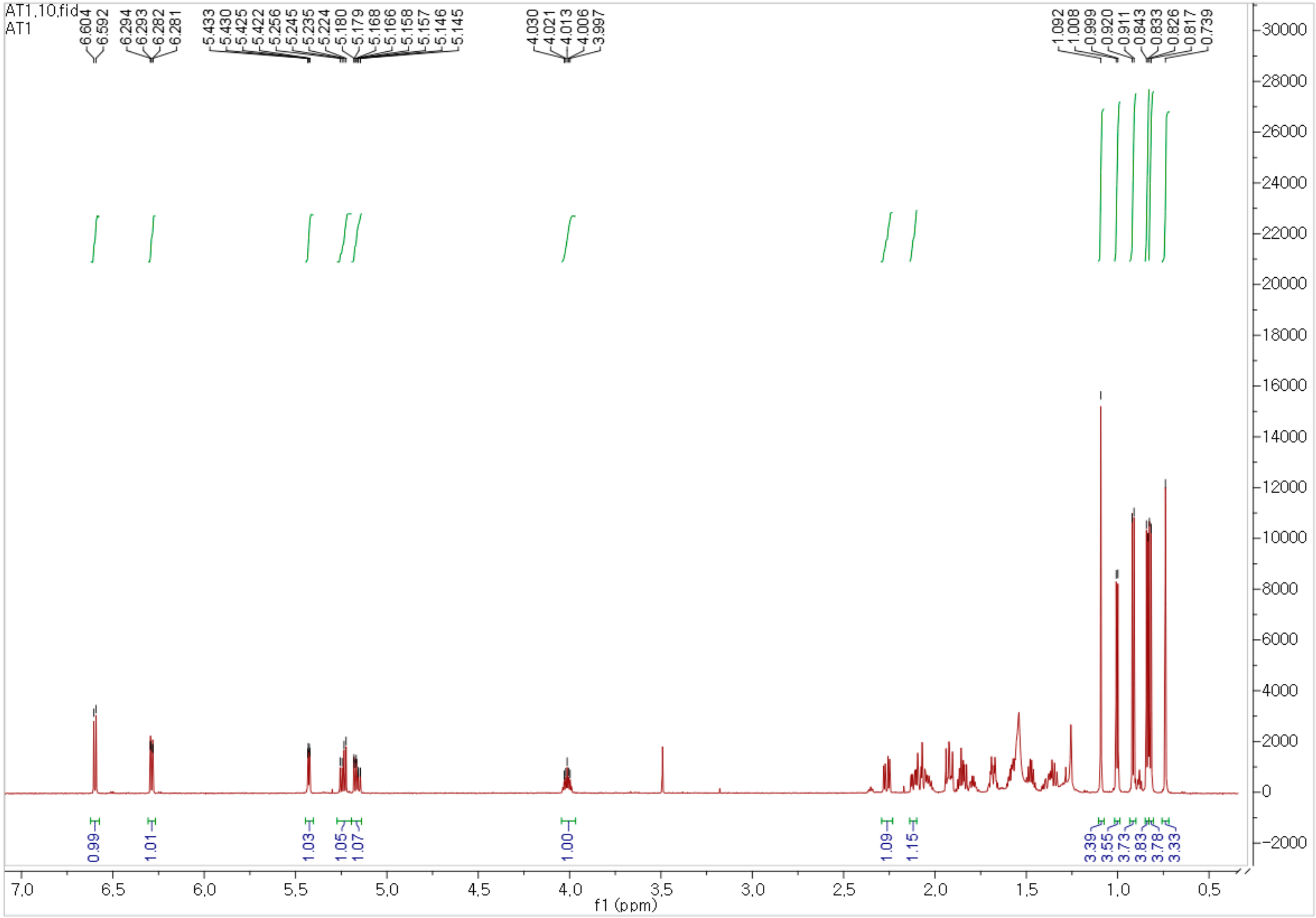

Figure S2. The ^{1}H NMR spectrum of **2** ($CDCl_3$, 700 MHz)

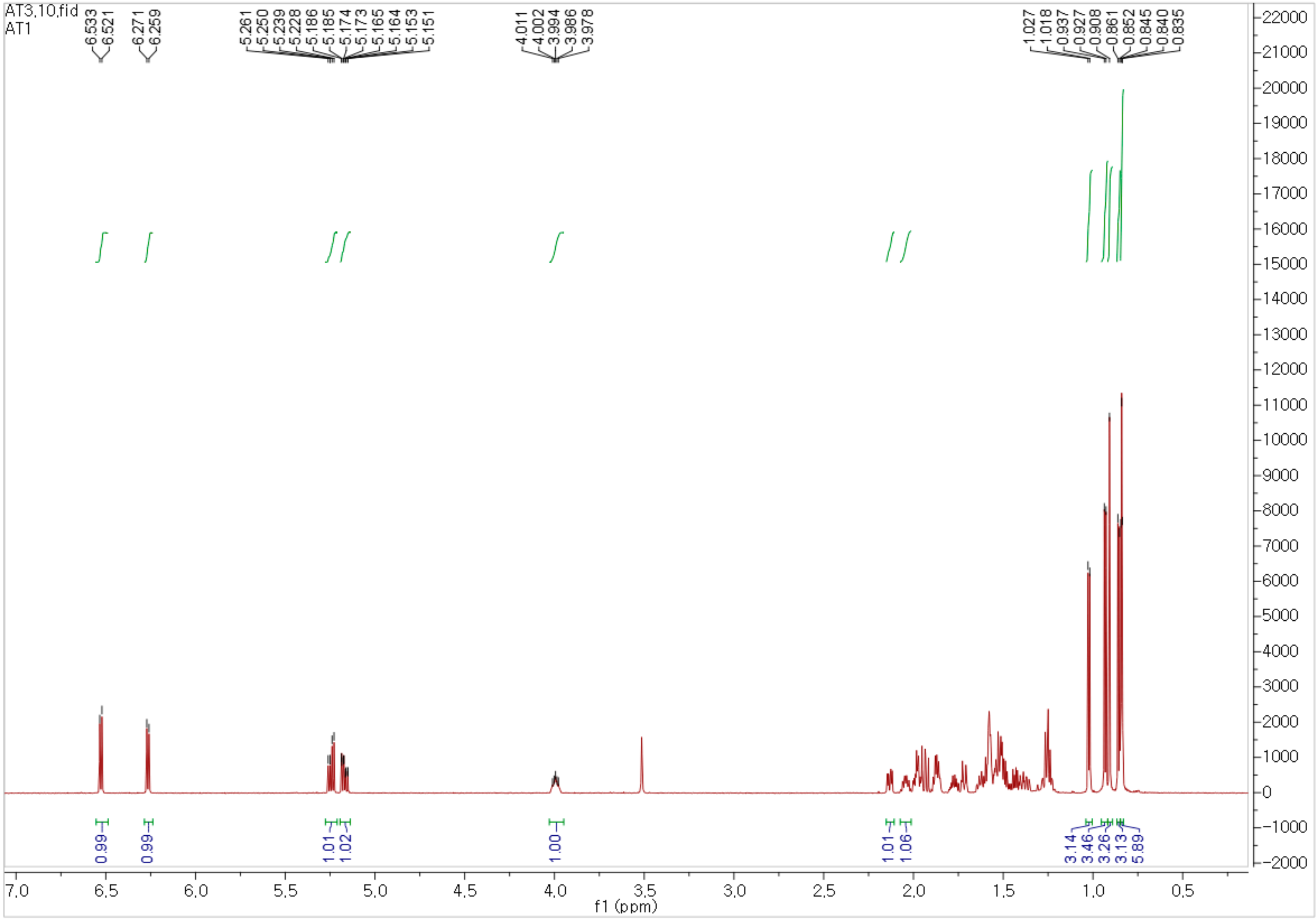

Figure S3. The ^{1}H NMR spectrum of **3** ($CDCl_3$, 700 MHz)

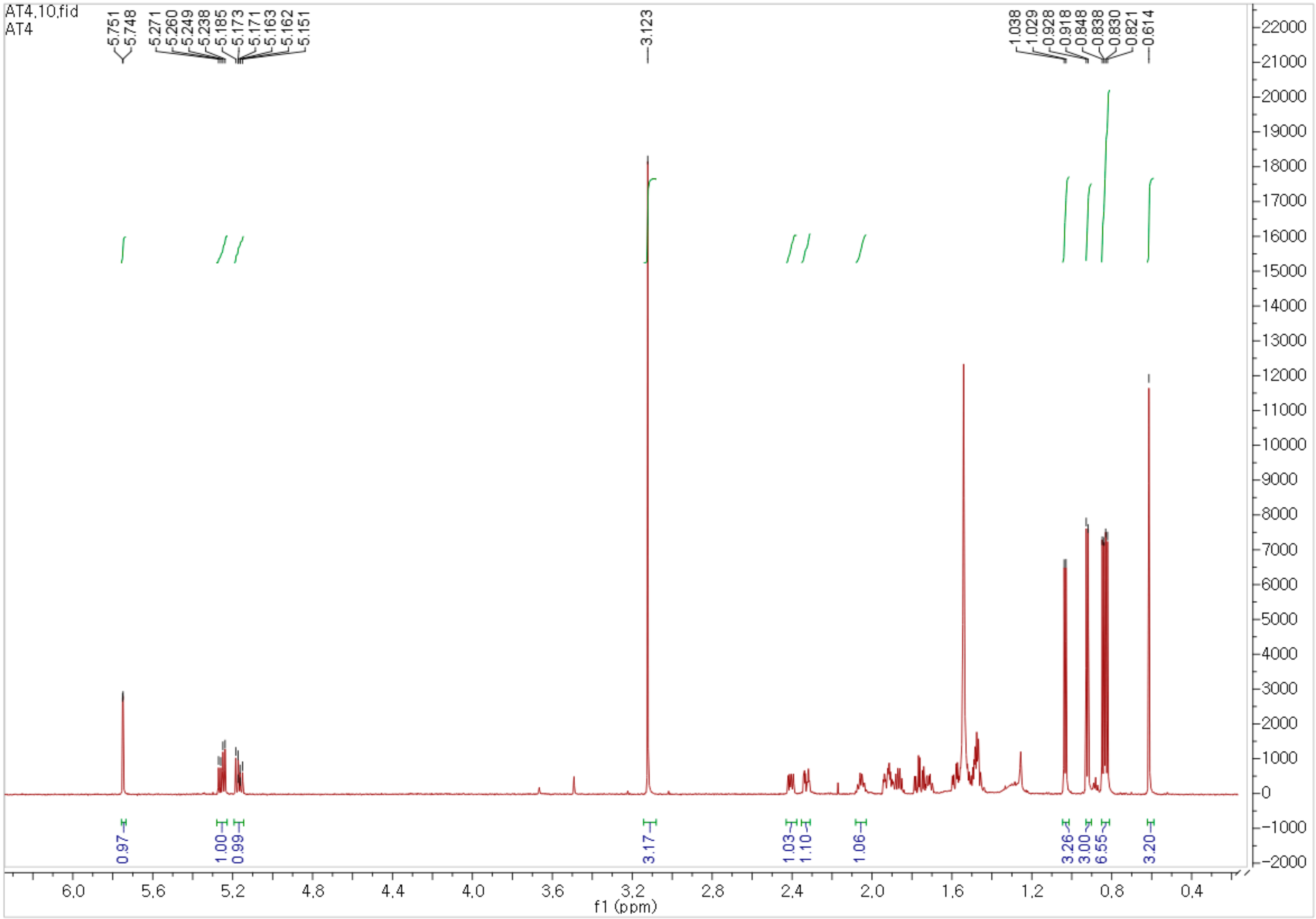

Figure S4. The ^{13}C NMR spectrum of **3** ($CDCl_3$, 175 MHz)

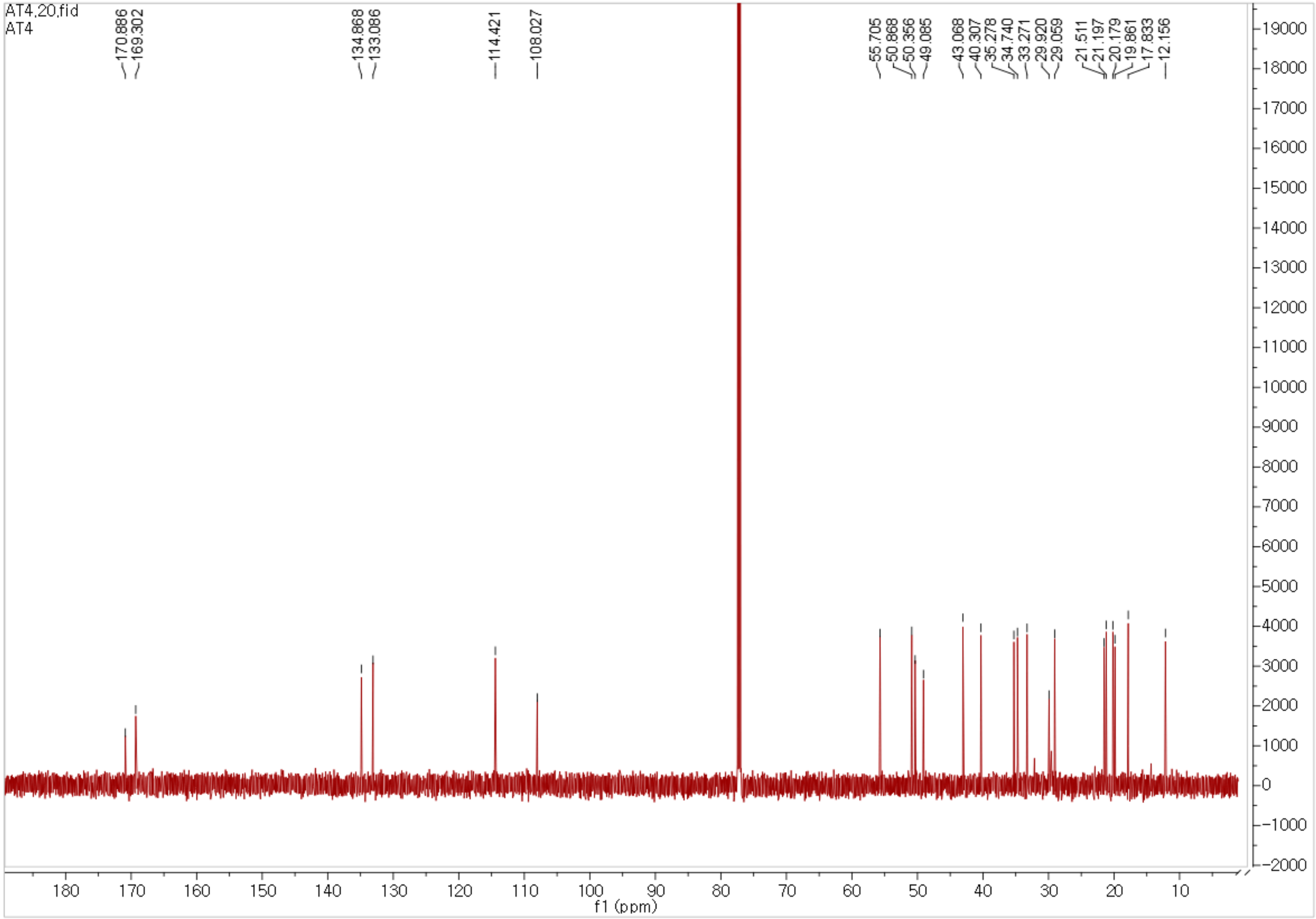

Figure S5. The ^{1}H NMR spectrum of **4** (CD_3OD, 700 MHz)

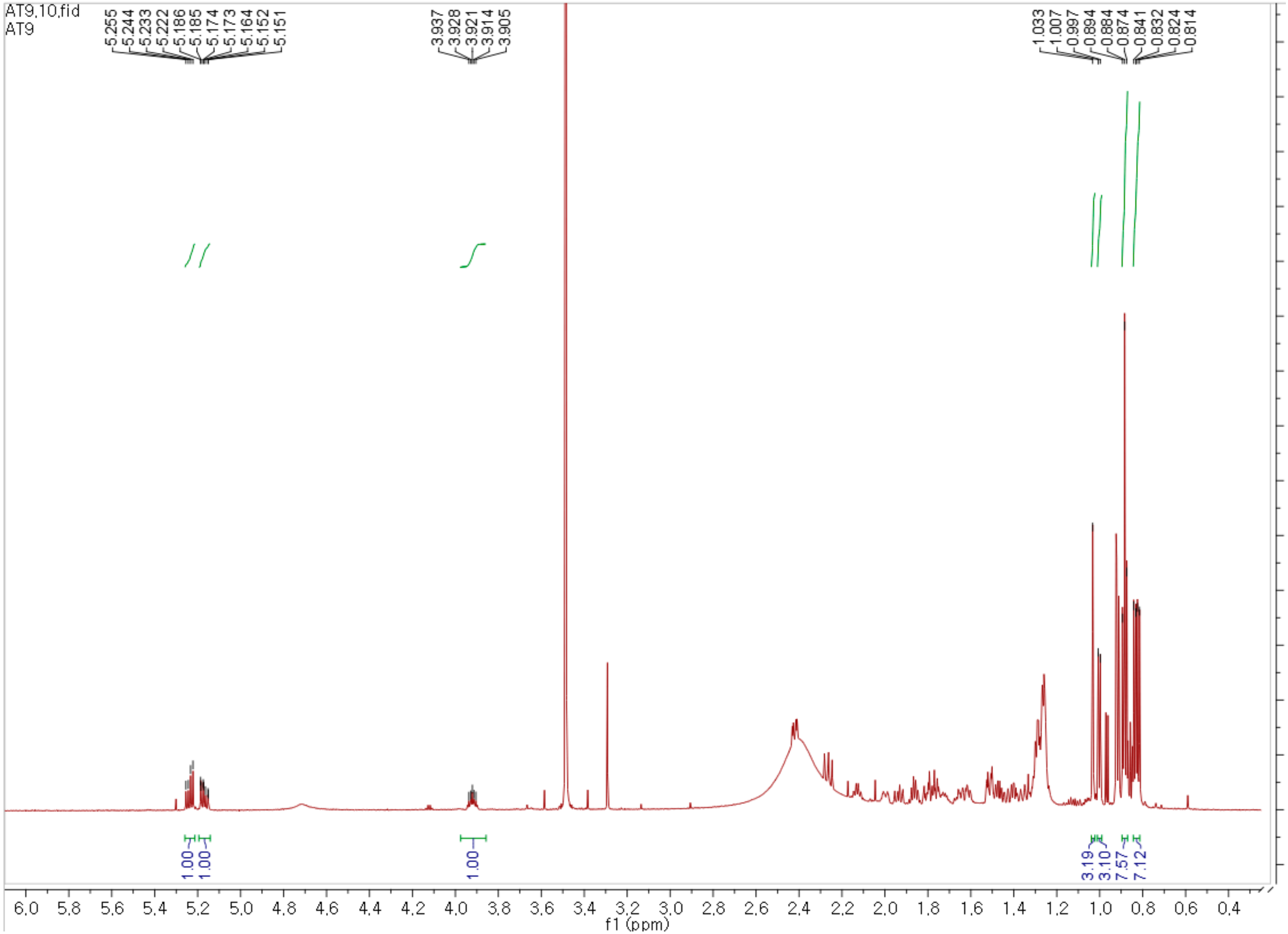

Figure S6. The ^{1}H NMR spectrum of **5** ($CDCl_3$, 700 MHz)

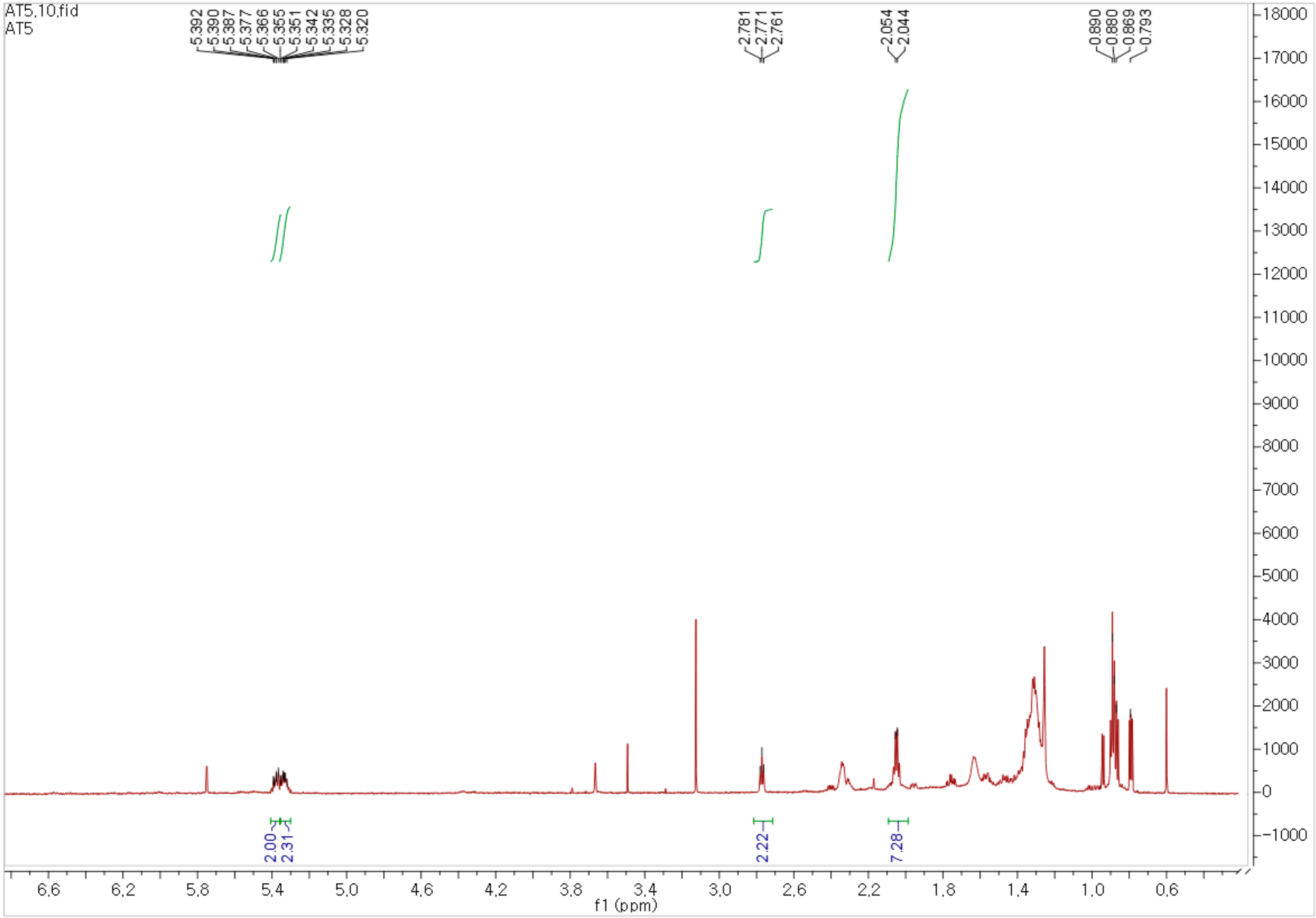

Figure S7. The ^{1}H NMR spectrum of **6** ($CDCl_3$, 700 MHz)

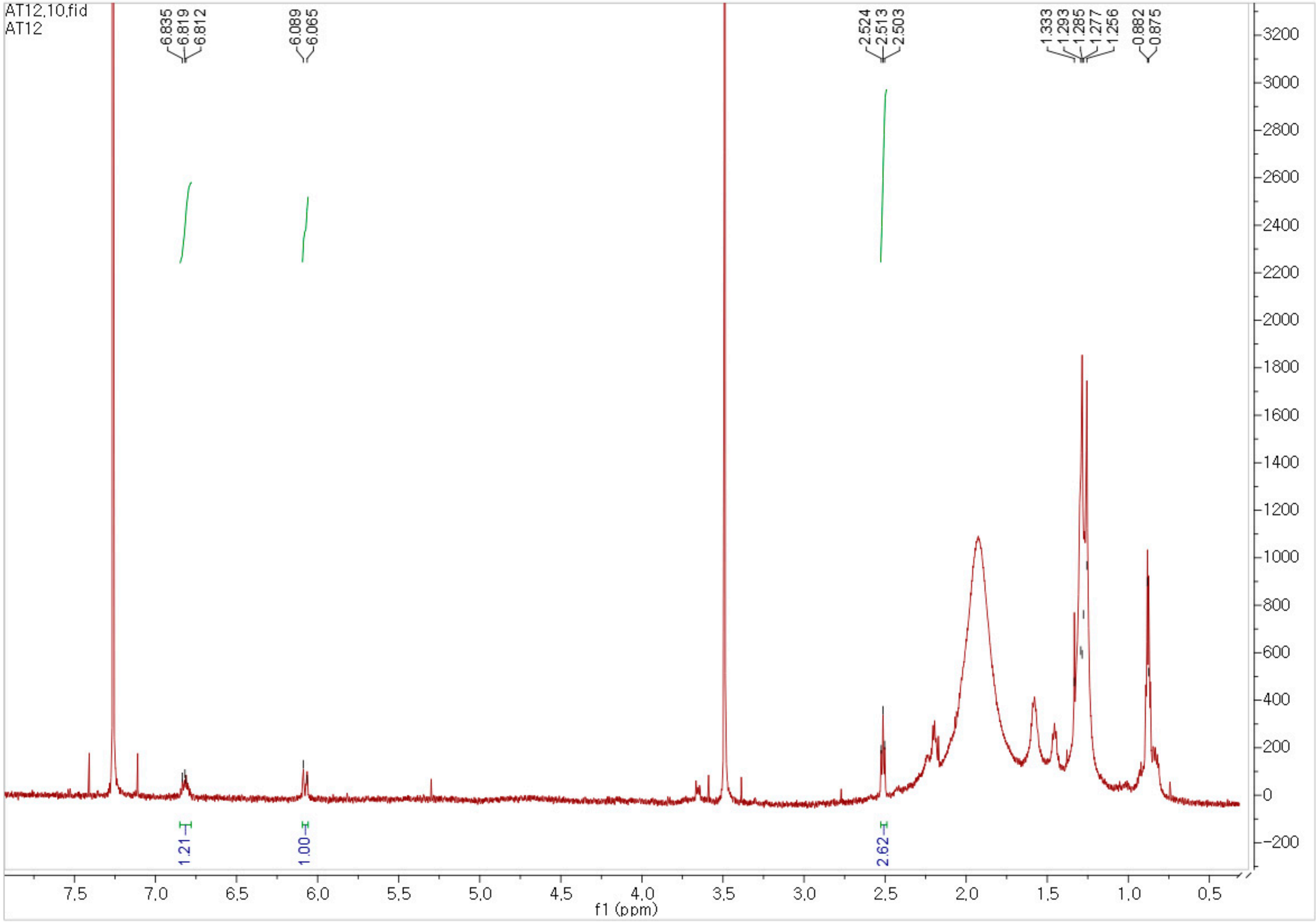

Figure S8. The ^{1}H NMR spectrum of **7** ($CDCl_3$, 700 MHz)

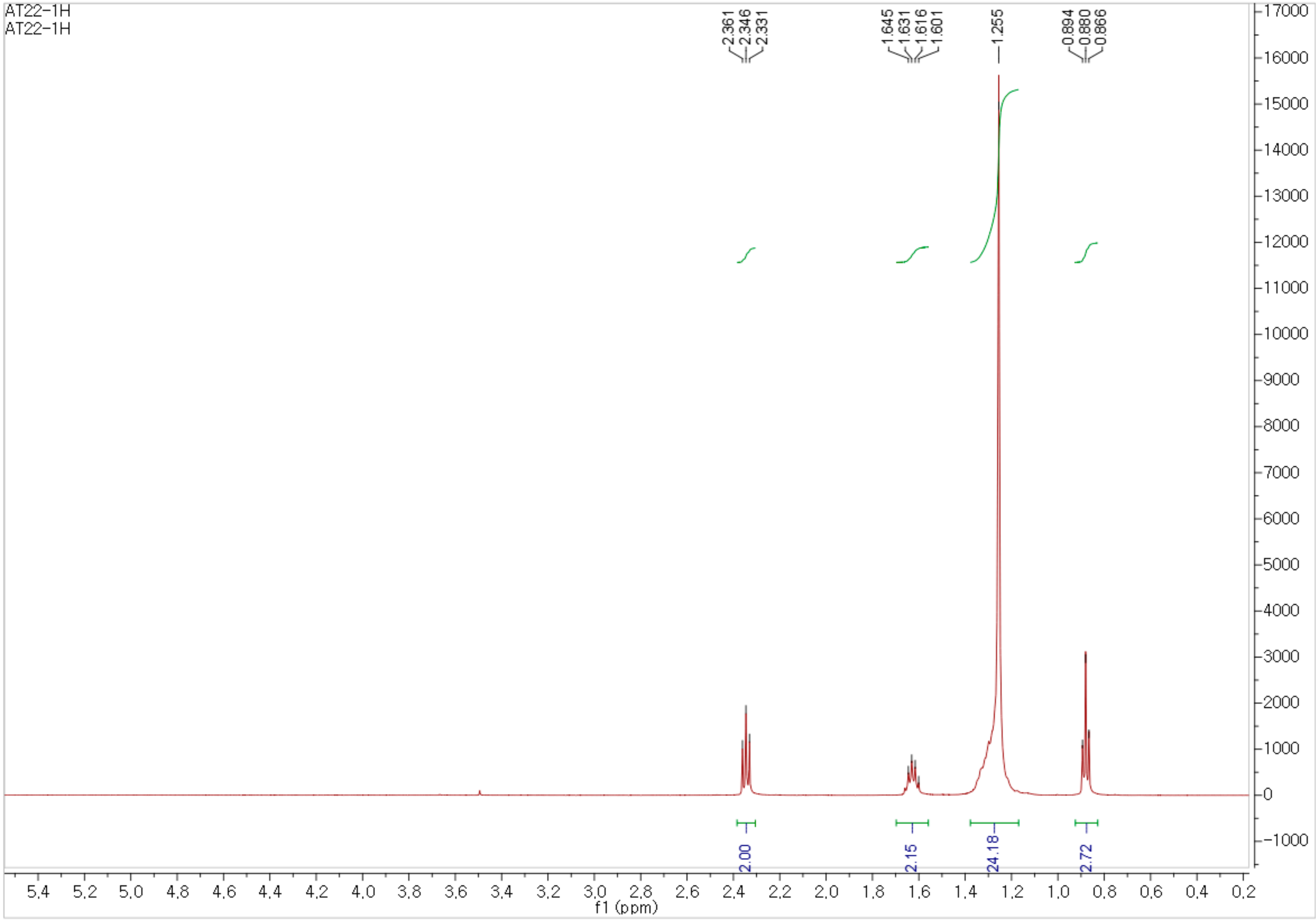

Figure S9. The ^{1}H NMR spectrum of **8** ($CDCl_3$, 700 MHz)

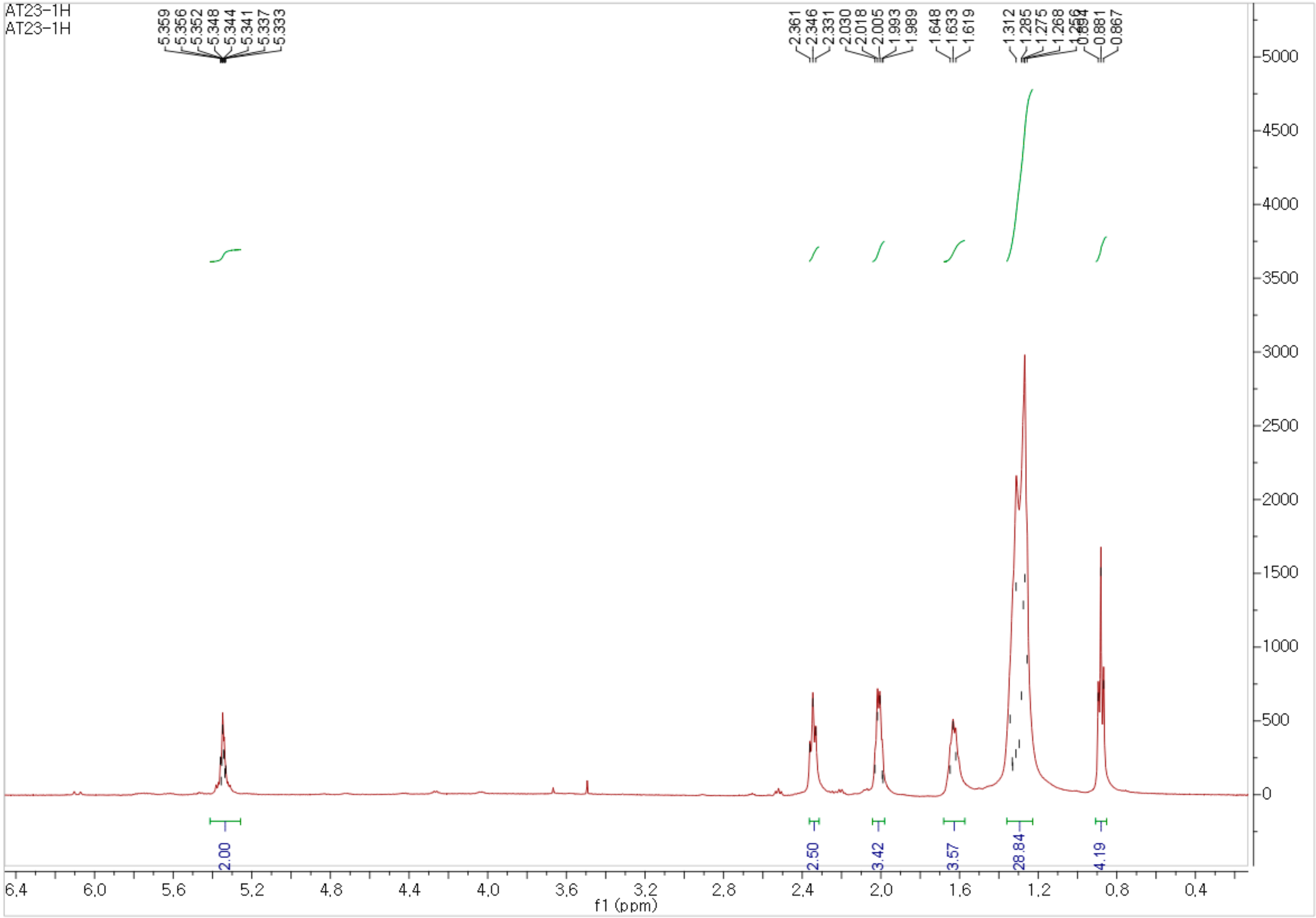

Figure S10. Total ion chromatogram (TIC) of compound **1** in the LC/MS analysis.

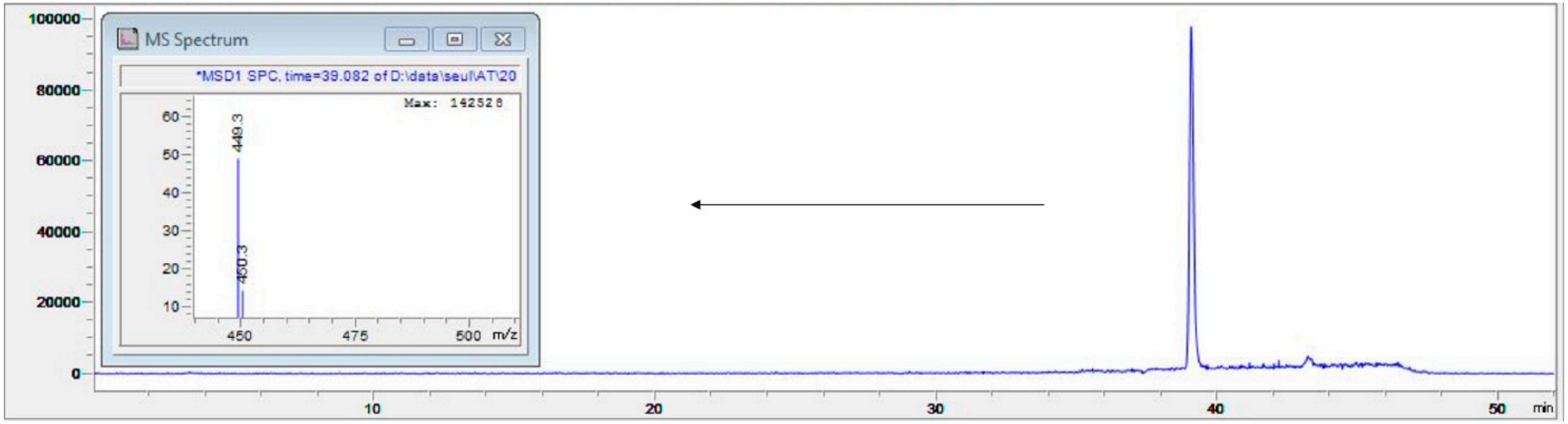

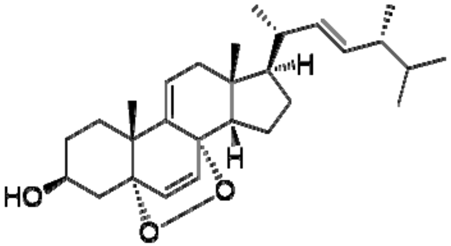

449.3 [M+Na]$^+$

Figure S11. TIC of compound **2** in the LC/MS analysis.

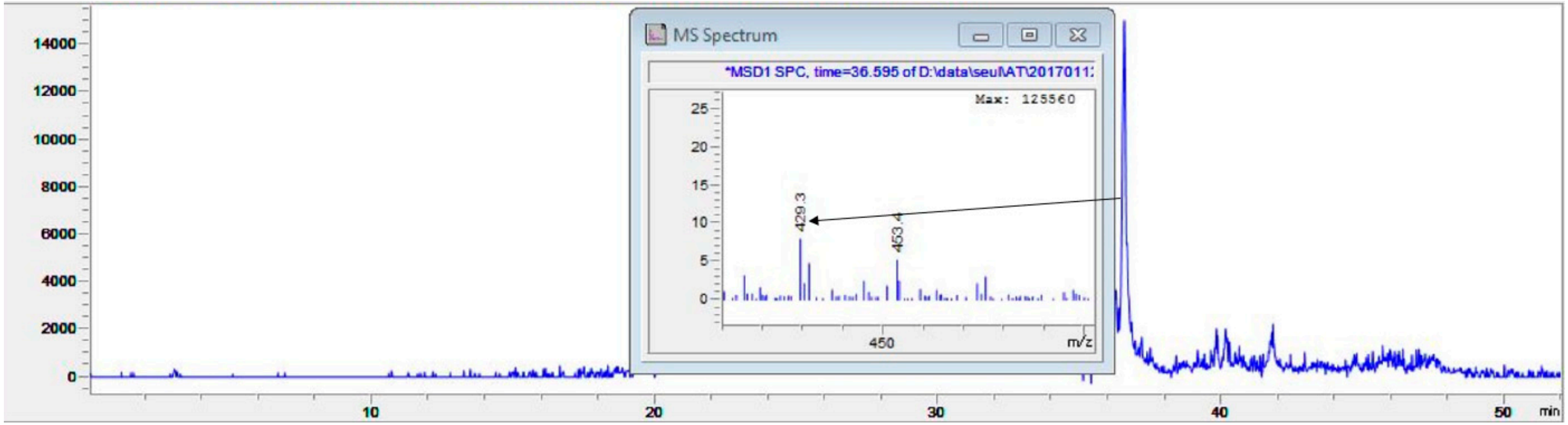

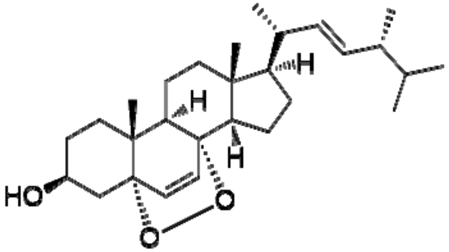

429.3 $[M+H]^+$

Figure S12. TIC of compound **3** in the LC/MS analysis.

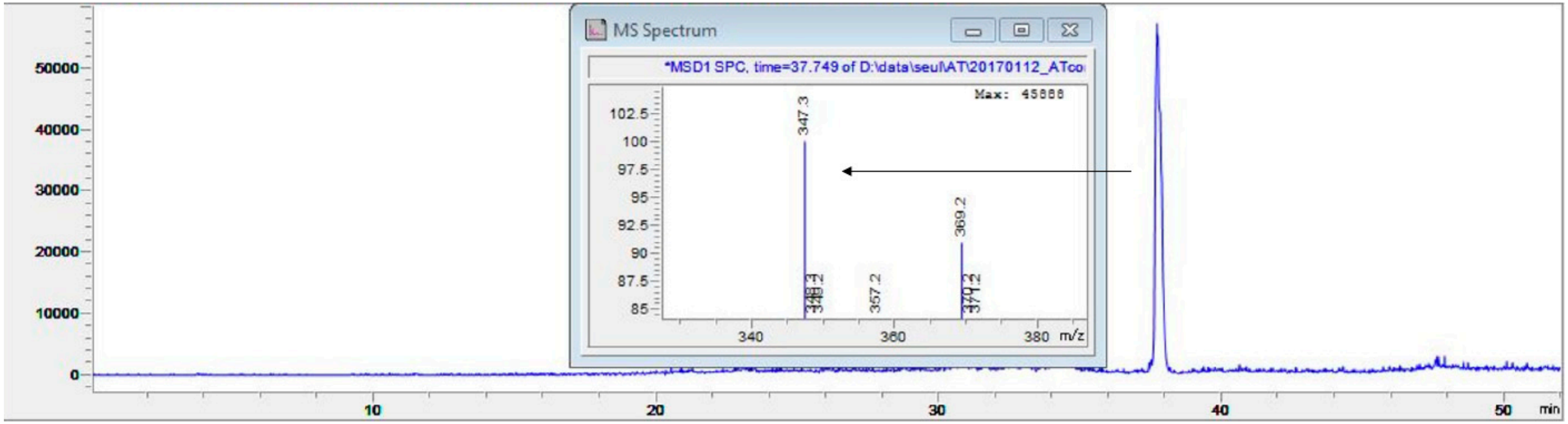

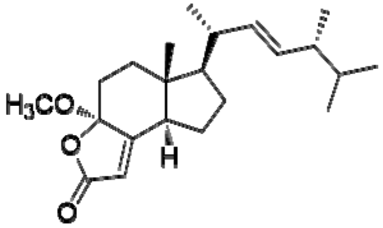

347.3 $[M+H]^+$

Figure S13. TIC of compound **4** in the LC/MS analysis.

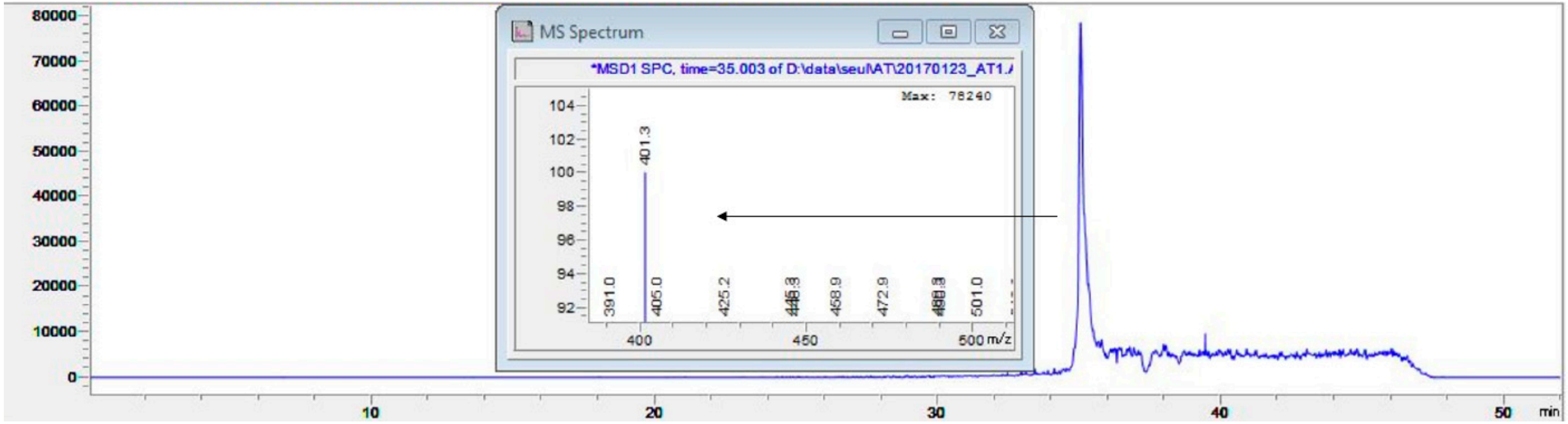

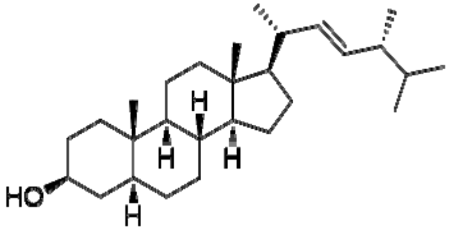

401.3 [M+H]+

Figure S14. TIC of compound **5** in the LC/MS analysis.

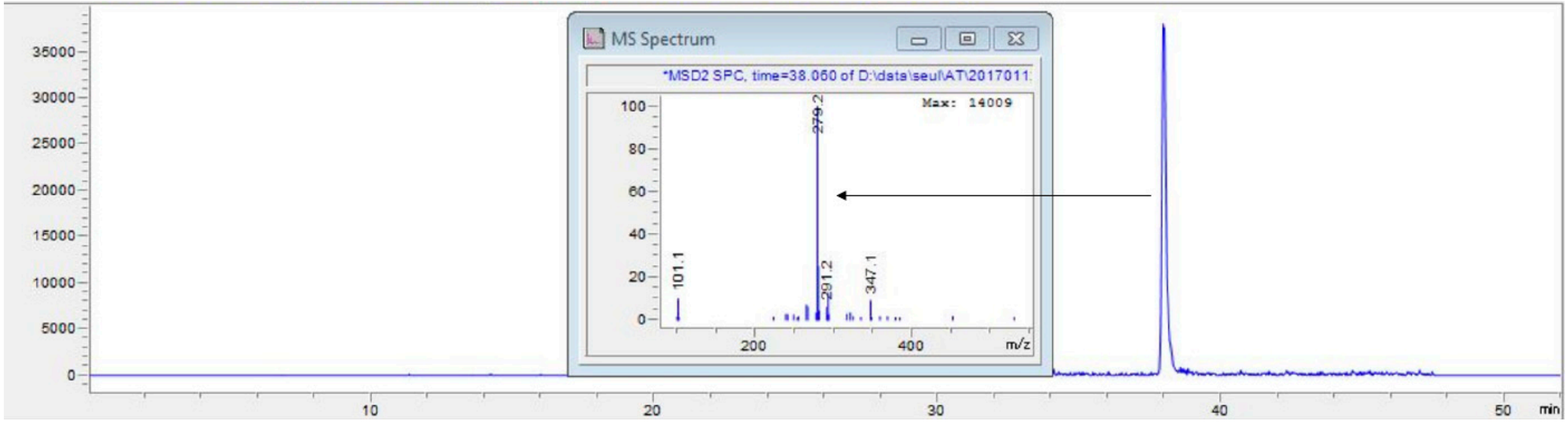

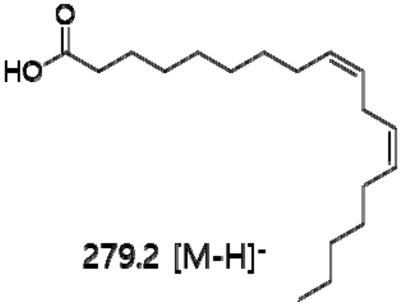

Figure S15. TIC of compound **6** in the LC/MS analysis.

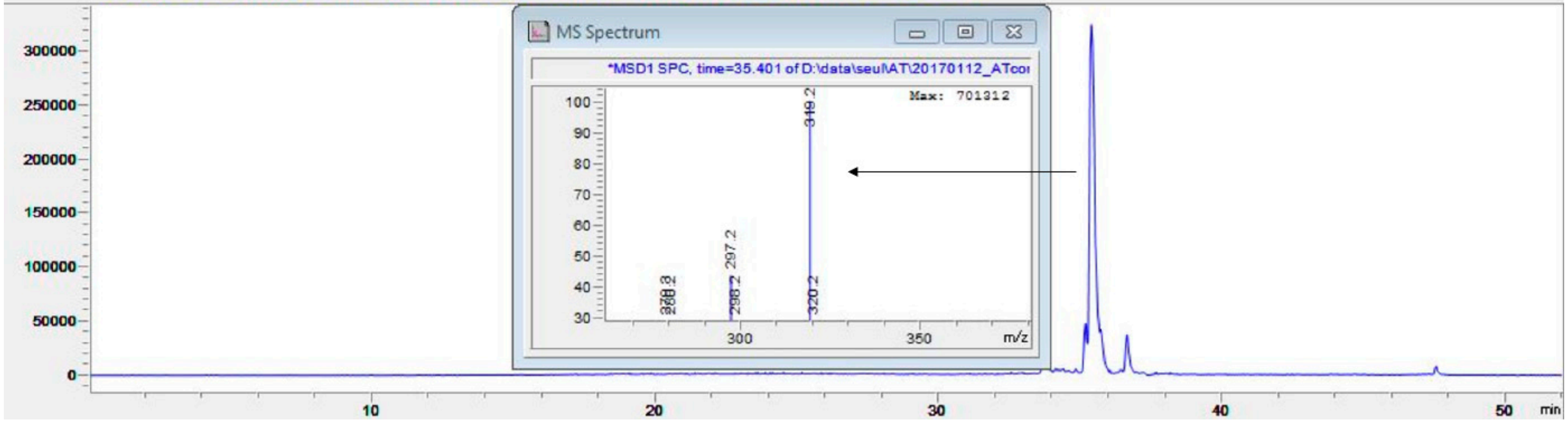

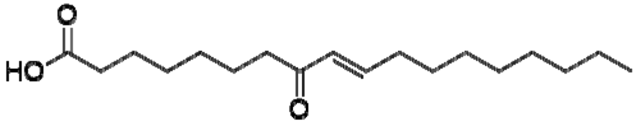

319.2 [M+Na]$^+$

Figure S16. TIC of compound **7** in the LC/MS analysis.

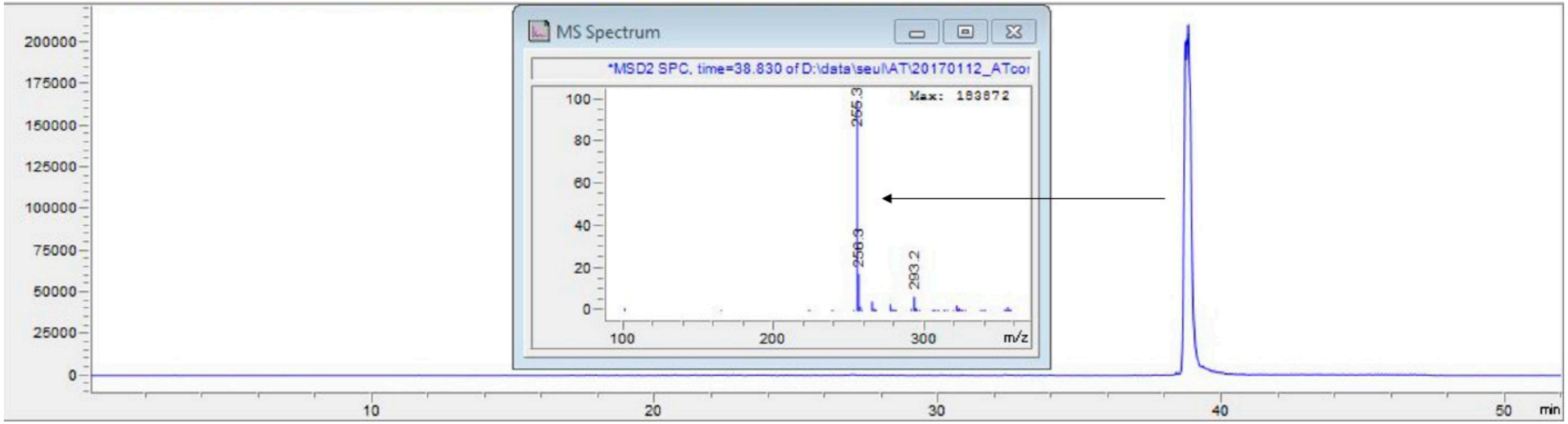

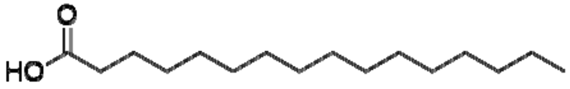

255.3 [M-H]$^-$

Figure S17. TIC of compound **8** in the LC/MS analysis.

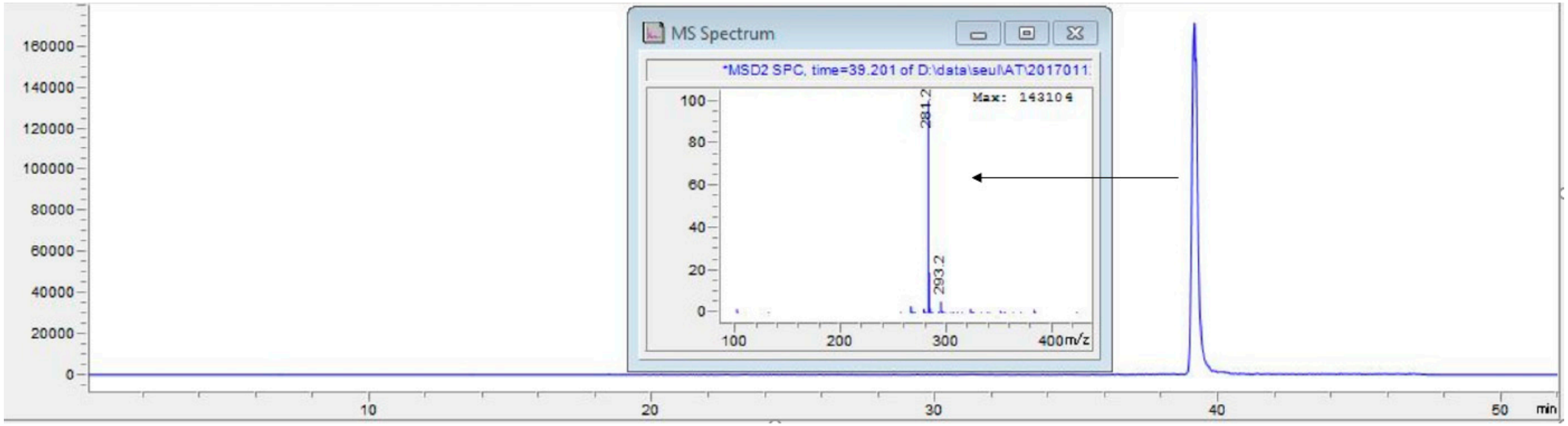

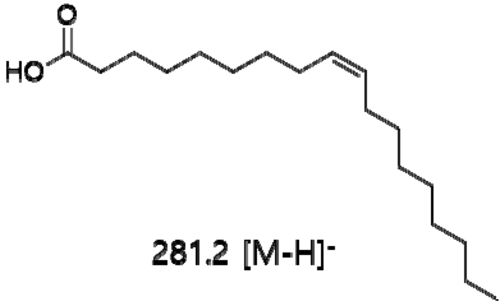

Figure S18. UV chromatogram of LC/MS (detection wavelength was set as 254 nm) of (A) hexane-soluble and (B) CH_2Cl_2-soluble factions.

(A)

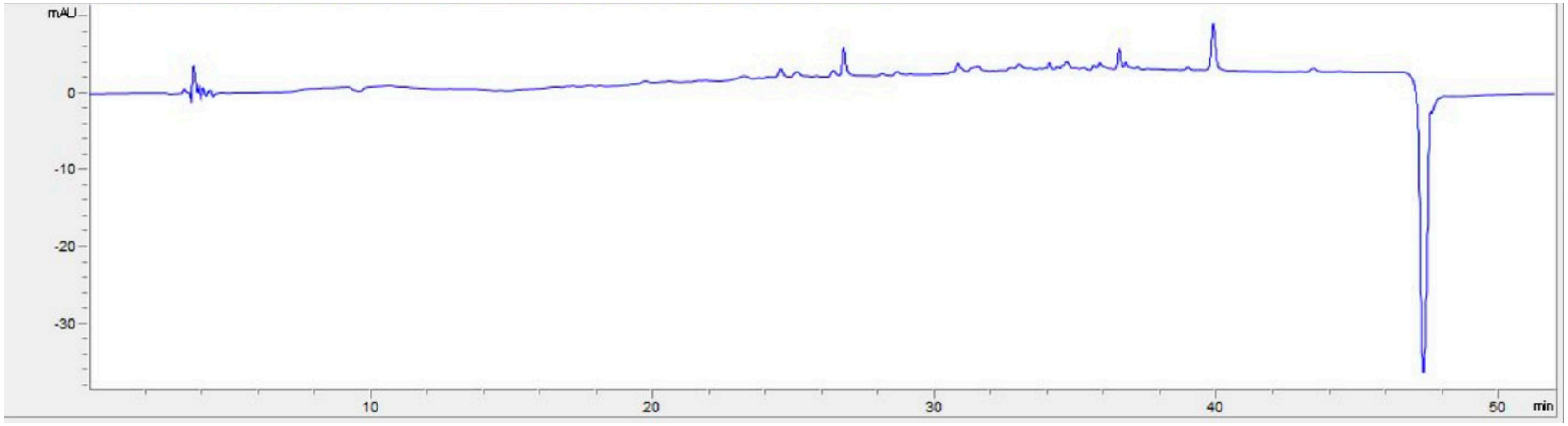

(B)

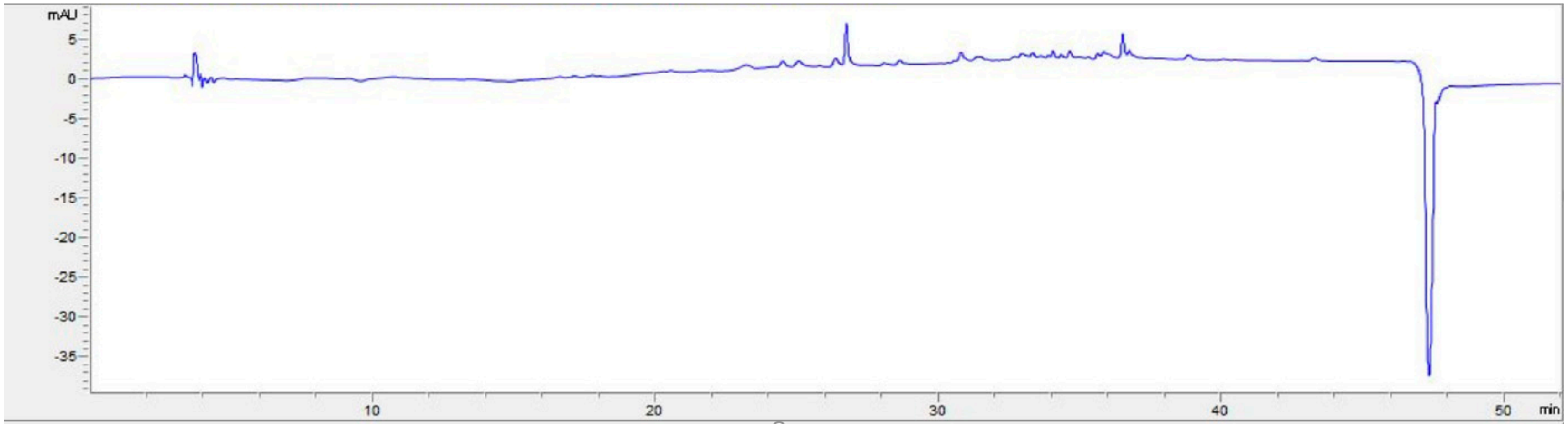

Figure S19. TLC analysis of (A) hexane-soluble and (B) CH_2Cl_2-soluble factions.

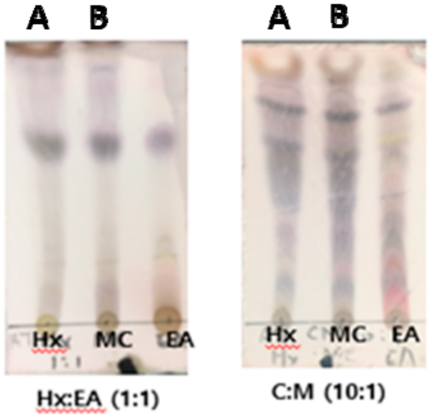

Supplement: Supplementary file 1 [file molecules-27-00036-s001.zip › molecules-1477228-supplementary.pdf]
